# The elimination of hepatitis D as a public health problem: Needs and challenges

**DOI:** 10.1111/jvh.13891

**Published:** 2023-10-03

**Authors:** Thomas Vanwolleghem, Paige A. Armstrong, Maria Buti, David FitzSimons, Sara Valckx, Greet Hendrickx, Pierre Van Damme

**Affiliations:** 1 Laboratory for Experimental Medicine and Pediatrics, Faculty of Medicine and Health Sciences, University of Antwerp, Antwerp, Belgium; 2 Universitair Ziekenhuis Antwerpen, Edegem, Belgium; 3 Viral Hepatitis Prevention Board, Centre for the Evaluation of Vaccination (CEV), VAXINFECTIO, University of Antwerp, Antwerp, Belgium; 4 Division of Viral Hepatitis, US Centers for Disease Control and Prevention (CDC), Atlanta, Georgia, USA; 5 Hospital General Universitari Valle Hebron, Barcelona, Spain; 6 Independent Researcher, London, UK

**Keywords:** diagnosis, epidemiology, guidelines, hepatitis D virus (HDV), public health, treatment

## Abstract

Infection with hepatitis D virus leads to liver disease and cancer most rapidly of all hepatitis viruses. However, knowledge about hepatitis D remains poor and the burden and impact are underestimated, even though some 12–15 million people mainly in low- and middle-income countries may be affected. Its epidemiology is changing, with increasing migration leading to increased risks of infection and disease. A recent Viral Hepatitis Prevention Board meeting reviewed the current epidemiological status, improvements in diagnostic testing, advances in the development of novel antiviral agents in phase III trials and the need for a greater public health response, such as new guidelines and recommended testing of all people newly identified as infected with hepatitis B virus for hepatitis D virus infection. It identified issues and needs for attention with regard to prevention, diagnosis and treatment.

Hepatitis D (HDV) is increasingly becoming recognized as a serious worldwide public health issue. Estimates of global prevalence are, however, incomplete and the epidemiology is shifting as a result of migration, improvements in diagnostic testing and novel antiviral agents in clinical trials. These changes present new opportunities for understanding and treatment.

The Viral Hepatitis Prevention Board aims to contribute to the control and prevention of viral hepatitis by raising awareness, issuing guidance on prevention, catalysing the development of recommendations and encouraging actions to reach these goals. It convened a group of experts in October 2021 to discuss the changing context of hepatitis D in order to identify the public health impact and to consider the inclusion of HDV infection in viral hepatitis elimination goals, such as those set by the World Health Organization (WHO) for 2030.^1^ Although hepatitis D was not included in WHO’s global health-sector strategy for viral hepatitis during 2016–2021, it is mentioned in the new strategy for the period 2022–2030, which was noted with appreciation by the World Health Assembly in May 2022.^2,3^ However, despite its inclusion in the newest strategy, there are no specific targets associated with HDV infection. Public health experts, virologists and clinicians identified a series of issues and needs for consideration and action (see Box: Issues and needs identified).

HDV replicates in the nucleus of hepatocytes and depends on the hepatitis B virus (HBV) for packaging and binding to liver cells. HDV and HBV coinfection (infection at the same time) results in acute hepatitis that can lead to fulminant hepatitis in a small proportion of cases but rarely leads to chronic infection. However, superinfection (acquisition of HDV in an already HBV-infected person or HBV carrier) usually leads to severe chronic infection and an accelerated progression to liver cirrhosis and end-stage liver disease, including decompensated cirrhosis and hepatocellular carcinoma.^4^ Risk factors for HDV infection are similar to those for HBV infection (injection drug use, commercial sex work and sex between men), but perinatal transmission is rare.

The burden and impact of HDV infection are underestimated owing to the limited availability of screening tests for anti-HDV antibodies, particularly in low- and middle-income countries where hepatitis D is more frequent. Identifying anti-HDV antibodies is the first step in the diagnosis of HDV infection, which needs to be followed by the detection of HDV RNA. Often, RNA detection is done by using in-house techniques, which are insufficiently standardized and validated. As a result, their use is restricted to academic centres. These factors have probably also led to an incomplete understanding of the epidemiology of HDV infection and to a lack of strategies for testing HDV in those communities with populations at risk.

The epidemiology of HDV is linked to the implementation of the hepatitis B vaccine. As HDV infection requires a concurrent HBV infection for HDV to propagate and as global coverage rates of universal childhood hepatitis B vaccination rise, reported incidence rates of HDV infection have declined in some regions. At the same time, rates of hepatitis B vaccination among adults remain low globally, and therefore the risk of HDV infection persists. Furthermore, gaps in hepatitis B vaccination coverage, exacerbated by the COVID-19 pandemic,^5^ risk expansion of the pool of individuals susceptible to HDV infection. Past outbreaks of fulminant hepatitis D have resulted in high mortality, and continuing spread of infection has been documented in unvaccinated cohorts.^6^ People living with chronic HBV infection are at risk of HDV infection and those with ongoing risk behaviour should be screened regularly for anti-HDV antibodies, and, if positive, for active HDV infection. Currently, there is no vaccine under development against HDV.

Although nationally representative estimates on the burden of HDV infection are scarce, global estimates suggest at least 12–15 million people are affected.^7,8^ Among people positive for hepatitis B surface antigen (HBsAg), the highest proportions infected with HDV are seen in the WHO’s African, South-east Asia, Eastern Mediterranean and Western Pacific regions.^5^ Countries and areas reporting the highest burden of HDV infection are Mongolia, Uzbekistan, Kyrgyzstan, the Punjab region of India, Pakistan and Somalia as well as the Amazon Basin in South America. In Europe, the disease burden significantly declined in the 1990s, but immigration from HDV-endemic countries has slowed that decline.^9^ In low- and middle-income countries, where HDV infection is endemic, there has been no substantial change over time in the epidemiology. In high-income countries, in contrast, the epidemiology has shifted from people in older age groups to younger persons, often migrants from countries where HDV infection is endemic. Recent studies performed in France showed that 86% of cases of hepatitis D occurred in migrants.^10^ Diagnosis of HDV infection requires the determination of HDV RNA, but the US Food and Drug Administration (FDA), for instance, has no approved assay for HDV RNA or for anti-HDV antibodies. HDV diagnostics are not included in WHO’s list of essential diagnostics. Testing algorithms typically involve testing all HBsAg-positive individuals for anti-HDV antibodies, and subsequent diagnosis of anti-HDV-positive individuals with HDV RNA by quantitative PCR. As anti-HDV antibody assays are not widely available there has been only limited standardization of HDV RNA PCR assays. Research into new assays is being done, in academic institutions and by laboratory companies.

International and national clinical practice guidelines vary in recommendations regarding HDV screening. Guidelines from WHO (2015)^11^ and the European Association for the Study of the Liver (EASL) (2017)^12^ recommend screening all HBsAg-positive people for anti-HDV antibodies. The American Association for the Study of Liver Disease (AASLD) proposes a risk-based approach, based on country of birth or risk behaviour, but this strategy has failed to identify most HBV/HDV-infected patients. With a high burden of infection among immigrant persons, specific strategies to reach populations likely to be disproportionately affected may be needed to improve health equity, as has been done for groups at higher risk for HCV infection.^13^

## Therapy.

Treatment options for hepatitis D are increasing. Until 2020, the only drug available for the treatment of HDV was pegylated interferon-alpha, and it is still recommended for off-label use with weekly injections for 48 weeks. About 25% of patients who receive pegylated interferon achieve persistent suppression of viral replication but remain HBsAg-positive. Its use is hindered by considerable side effects and poor tolerance.^14^ Advances in virology are contributing to the development of new treatments for HDV infection. Bulevirtide (BLV), a first-in-class entry inhibitor of HBV, received conditional approval from the European Medicines Agency in 2020.^15^ BLV is administered at a dose of 2 mg subcutaneously daily in patients with compensated chronic hepatitis D. At 48 weeks of therapy, 45% of those receiving BLV achieve a >2 log decline in the levels of HDV RNA or undetectable HDV RNA. The treatment is well tolerated and the main side effect is a transient increase in serum bile acid levels.^16^ BLV is now under evaluation in a phase III study. In addition, real-world data from different cohorts of patients with compensated liver disease treated with BLV show similar results to those observed in the registration studies. BLV is administered as long-term monotherapy daily or in combination with pegylated interferon for a finite duration. However, the drug is only available and reimbursed in a few EU countries such as France, Italy and Germany and has recently been approved in the UK as well. In addition to more data on therapy duration, we need predictors of response to BLV and guidelines for stopping treatment in order to better define the best therapeutic strategies. Another drug under development is lonafarnib, a small non-peptide molecule and prenylation inhibitor targeting the host enzyme farnesyl transferase which is used by HDV assembly. Lonafarnib, administered orally, is approved by the FDA for the treatment of rare disorders. It is currently under investigation for the treatment of hepatitis D, either in monotherapy or in combination with pegylated interferon in a large cohort of patients. Preliminary results look very promising.^17^ Other drugs are interferon-lambda, which is in a phase III clinical trial to assess whether it is better tolerated than other interferons, and a nucleic acid polymer REP2139-Ca, which inhibits the release of subviral hepatitis B particles from infected hepatocytes and is also in trials.^18^

The most appropriate endpoints for the treatment of hepatitis D are still controversial. EASL/AASLD agreed in a special conference on two HBV/HDV endpoints.^19,20^ First, a finite strategy requires HDV RNA to be undetectable off-treatment, with normalization of alanine aminotransferase (ALT) activity. Secondly, a maintenance strategy requires an on-treatment reduction of >2 log for HDV RNA with ALT normalization. Although the first is an acceptable endpoint in clinical cohort studies, the latter lacks outcomes for clinical validation.

## Hepatitis D is a public health issue.

Overall, public health responses to hepatitis D are lacking or unremarkable. Data on the burden of HDV infection, inadequate screening and suboptimal treatment options are inadequate. This lack contributes to poor knowledge and awareness of hepatitis D among the general population, public health professionals, clinicians and policy-makers alike. All participants agreed that reflex testing for anti-HDV should be done in all new HBsAg-positive cases and incorporated into the management guidelines and policies. This will increase the detection and early diagnosis of new cases of HDV infection. Expanded testing would not only improve clinical outcomes and prognosis for individuals screened but also contribute to a more comprehensive understanding of the burden and global epidemiology of HDV infection. Advocacy and education campaigns and further public health and clinical research, along with the publication of data would help to raise awareness. New guidelines, specific to HDV, would be useful to guide and educate the scientific community, healthcare workers, patients and policymakers. In particular, healthcare workers need to be educated about prevention, control and management of HDV infection. The importance of HBV vaccination needs to be highlighted with specific examples (such as the success of the hepatitis B vaccination programme in Peru,^21^ which reduced not only the number of cases of HBV infection but also the burden of HDV infection).

In summary, the VHPB’s technical meeting increased awareness about hepatitis D virus infection, an essential step to control the infection. This effort has to be done together with HBV elimination. The Box below summarizes the issues and needs identified during the meeting.

## Data Availability

Data sharing not applicable to this article as no datasets were generated or analysed during the current study.

## Box. Issues and needs identified during the Viral Hepatitis Board meeting (October 2021)

- Definition of markers of infection and their dynamics (e.g. the probability of finding HDV antibodies at all stages of the clinical spectrum is low).
- Descriptions of the natural history of hepatitis D.
- Determination of the attributable fraction of HDV to cirrhosis and hepatocellular carcinoma (HCC) (estimated at 20%).
- Standardization of sera and reference materials.
- Development or refinement and standardization of diagnostic assays, including commercial, in-house and new tests (for instance, PCR tests for genotypes), that are available and affordable.
- Staging of liver fibrosis: development of reliable non-invasive tests.
- Conclusion of clinical trials of new therapies and investigation of new combination treatments, aiming for licensure and negotiations for availability and affordable pricing.
- Identification of reproducible virological endpoints that predict long-term clinical outcomes in clinical trials.
- Indications of when to stop treatment and evaluation of benefits of long-term versus short-term treatment.
- Development of alternatives to daily subcutaneous injection of bulevirtide such as an oral formulation or possibly self-administered injections.

## Abbreviations:

AASLD: American Association for the Study of Liver Disease
ALT: alanine aminotransferase
BLV: Bulevirtide
EASL: European Association for the Study of the Liver
FDA: US Food and Drug Administration
HBsAg: Hepatitis B surface antigen
HBV: hepatitis B virus
HCC: hepatocellular carcinoma
HDV: hepatitis D virus
VHPB: Viral Hepatitis Prevention Board
WHO: World Health Organization
